# The British Journals: Surgery

**Published:** 1840-04

**Authors:** 


### SURGERY.

*On the Treatment of Varicose Veins.* By A. T. S. DODD, Esq., Surgeon of the Chichester Infirmary.

[THIS is a valuable practical paper, and forms an appropriate *English* appendix to the Essay of M. Bonnet in our last Number. According to the views of this surgeon, it will be necessary for Mr. Dodd hereafter to ascertain the *permanency* of his cures, before the full value of the practice is admitted.]

At the Chichester Infirmary, during the last twelve months, eleven cases have been treated by the insertion of one or more needles under the vein, and the application of the twisted ligature. Of these, ten have been entirely successful in remedying the varicose state of the veins, and healing the accompanying ulcer. One was unsuccessful, the varicose state of the superficial veins being cured by the obliteration of those that were so affected, but the ulcers still continuing. In three cases troublesome symptoms occurred from the application of the needles. In one of these, the patient complained of severe pain in the sole of the foot. In the second, ulceration followed the introduction of the needles, but the cure was ultimately complete. In the third, inflammation of the veins occurred, which, however, readily yielded to treatment.

The following is a summary account of the eleven cases:

| No. | Name. | Age. | Duration of the Ulcers. | No. of Needles Inserted. | Result. | Duration of Treatment. |
|---|---|---|---|---|---|---|
| 1 | John Downham | 49 | 16 years | 5 | cured | 7 weeks |
| 2 | Eliza Luffe | 33 | 9 years | 1 | cured | 7 weeks |
| 3 | Do., readmitted for an ulcer on the other leg | 33 | 9 years | 2 | cured | 5 weeks |
| 4 | Redman Mary | 59 | 20 years | 1 | cured | 5 weeks |
| 5 | Henry Herman | 49 | 15 years | 1 | cured | 4 weeks |
| 6 | Wm. Barnes | 44 | many years | 5 | uncured | |
| 7 | Richard Dyer | 55 | 2 months | 3 | cured | 3 months |
| 8 | Sarah Luffe | 28 | 1 month | 2 | cured | 4 weeks |
| 9 | Martha Puddick | 38 | 13 years | 2 | cured | 7 weeks |
| 10 | Sarah Pullen | 58 | no ulcer | 5 | cured | 4 weeks |
| 11 | James Dawson | 52 | several years | 2 | cured | 5 weeks |

The plan of treatment which is the subject of this communication was first recommended, as far as I know, by M. Velpeau. It consists in passing a needle (I prefer a flat one, slightly curved at the point, such as is used in inspections,) through the integument, a little to one side of the vein, taking care that it shall go under the vessel without wounding it, and bringing it out at the same distance on the opposite side. A waxed silk ligature is then passed round the projecting ends of the needle, in form of a figure 8, just as it is applied in hare-lip. The projecting point of the needle is guarded by a little bit of cork, to prevent its catching in the clothes, or injuring the other leg. Very slight pressure is sufficient to accomplish the object to be gained by the ligature, therefore it need not be drawn tighter than is necessary to obstruct the circulation through the vein. In about from twenty-four to thirty-six hours, inflammation is excited in the immediate neighbourhood of the ligature, which goes on increasing slowly while the needle remains. As this inflammation is the effective means of cure, by producing permanent obstruction to the flow of blood through the vein, and obliterating the cavity of this vessel, it is obvious that it should be allowed to proceed just so far and no further than is necessary for the accomplishment of this object, and that the length of time which the needle is allowed to remain must be entirely regulated by this. My colleague, Mr. Duke, and I have generally found that from four to eight days have been sufficient to effect our object, though the exact time must of course vary according to the nature of the individual constitution and its aptitude in taking up the inflammatory action. I am guided by the appearance of the local inflammatory symptoms, and by the fact of the commencement of ulceration at the point of the skin where the needles pierce it. If this has begun, it is time to remove the needle, and, generally speaking, enough inflammation is found to follow for the sealing up of the vein. If ulceration has not taken place when the needle is withdrawn, it is generally necessary to repeat the operation, from the circulation being renewed through the diseased vessel. At the same time it is necessary to be careful not to allow the process of ulceration round the needle to remain in action long, as from this cause, in one case, troublesome ulcers resulted in this very spot, though the sore for which the practice was adopted was cured. I have not found this to occur when the above precaution was adopted. The only adjuvant treatment that has generally been required is low diet, recumbent position, and, when the needles are withdrawn, the application of the warm-water dressing. Should the inflammation appear more than needful, of course the treatment would be modified as circumstances arise.

The time required for the cure must of course vary much, according to the peculiarities of the case, from the habit of the individual, or the nature of the disease. In all the cases, but one, that we have treated, there was an ulcer connected with the diseased veins, but the duration of the treatment seemed to be not at all influenced by the previous duration of the ulcer, or indeed of the diseased state of vein, as will be seen by reference to the table. If the system bore the operation kindly, and too great irritation and inflammation was not set up, the cure generally proceeded rapidly, and was always effectual and permanent, if the treatment was carried to a proper extent. The shortest period required in any of our cases for the completion of the cure was three weeks, the longest seven weeks. The one case, which was of simple varicose veins, without an ulcer, had them immensely enlarged, and acutely painful and tender, and apparently ready to burst, so that the patient was unable to attend to her duties. Four weeks were here sufficient to make a permanent cure, the affected veins being reduced to a hard cord.

With regard to the number of needles necessary to be applied, though one was sometimes found to accomplish the obliteration of the vein, yet it was found a more effectual plan, and not productive of more after-suffering, to insert two under the same vein, at about two inches distance apart, though it is not always that the enlargement of the vein offers sufficient length for this. Where, however, it does, the obstruction produced by the two needles is more certain, and more effectual, without any greater risk, as far as my experience goes, of injurious effect to the vein. In some cases the ulcer does not yield, or yields only partially, to the application of the remedy to one vein, and then it is necessary to take up a second, or even a third, found in its vicinity. In one instance an enormous ulcer was rapidly reduced to about a fourth of its size by the obliteration of one vein; but though five needles were at different times inserted, and effectually, as regards the obstruction of the veins, yet the ulcer was never effectually cured. In the case of varicose veins, unaccompanied by ulcer, Mr. Shute, the house-surgeon, under whose care it was, inserted at the same time five needles—two upon the principal branch, and three others upon branches in the immediate neighbourhood, which communicated with it. Some erysipelatous inflammation was excited, which readily yielded on the removal of the needles, on the eighth day. The number of needles, however, which we employed in most of our cases was two.

The rapidity with which the curative effect upon the ulcer displayed itself was surprising. In twenty-four hours the commencement of cicatrization was seen in one instance, and in forty-eight hours the tender cuticle had more than half covered the granulations. This was not, however, so strikingly the case in every instance, though in all the progress was satisfactory. I observed, however, that the latter stages of healing were not commensurate with the earlier, in the progress of cure being much slower, and, in one instance, remaining stationary, after being readily reduced to about a fourth of its former size.

I was struck with the appearance of the cicatrix in all these cases. Instead of the reddish tender-looking cicatrix which we usually find after a recently healed ulcer, I observed that after the application of the needles the resulting cicatrix had, in the course of a few days after its formation, the firm whitish appearance of one of considerable standing, and even a scar of an old wound, in the neighbourhood of the ulcer under treatment, had its appearance modified, in becoming clean and free from a scaly incrustation, and assuming the white fine appearance of a healthy cicatrix.

*Med. Gazette. Dec. 20, 1839.*

*On Thread Setons.* By JONATHAN OSBORNE, M.D., Dublin.

THE ordinary seton, although a measure of great value when it is desired to keep up a permanent counter-irritation, is yet often attended with much unnecessary pain and inconvenience. Especially in the nape of the neck, the size of the strap of threads or of gum elastic introduced into the opening is productive of general discomfort and teasing, not well calculated to diminish a tendency of blood to the head, but rather the contrary. I have adopted a plan which will be found better suited to many of such cases. It is to make a seton with an ordinary sewing-needle of the thickest kind, and one thread of oiled silk. This is passed through a piece of the skin held between the finger and thumb, about six inches of the thread being allowed to remain. In twenty-four hours considerable redness comes on, and in a few days a purulent discharge is set up, much more in quantity than a comparison between the size of it and of the ordinary seton might lead to expect. The opening gradually enlarges, and no doubt in process of time, like the perforations made in the ears for ear-rings, assumes the function and secretion of a mucous membrane. The trifling degree of pain, however, inflicted by the operation enables us to multiply those setons, and to substitute new for old ones; so that I think it is evident, that in this way a greater discharge and a more efficient counter-irritation may be maintained, with less inconvenience than by the ordinary setons, and in places where the former would be impracticable.

*Dublin Journal. Jan.* 1840.

---

*Observations on Diffuse Inflammation.* By HENRY KENNEDY, M.B.

THIS is an elaborate essay on a very important subject. We regret that we have only room for the conclusions which Dr. Kennedy considers as warranted by the investigation entered upon by him. They are as follow:

1. That diffuse inflammation will not attack a person in perfect health.

2. That the bad state of health preceding diffuse inflammation is powerfully caused by anxiety of mind, by great bodily fatigue, by shocks of the nervous system, by improper diet, or by anything which has a tendency to lower the general healthy tone of the system.

3. That this deranged state of the health is shown principally in a vitiated state of the bowels.

4. That when once this unhealthy condition is established, the slightest cause is capable of inducing diffuse inflammation.

5. That venous inflammation does not necessarily cause diffuse inflammation.

6. That venesection may cause diffuse inflammation, the vein, however, remaining healthy.

7. That when venous inflammation does exist, the fever which accompanies it is more likely to be of the typhoid type than when diffuse inflammation exists alone.

8. That diffuse inflammation may attack several parts of the body in rapid succession, or it may be confined to one part, as the hip, or one organ, as the lung.

9. That pus may be poured out into the joints, serous cavities, or cellular structure, without any appearance of surrounding inflammation.

10. That, at the very onset of the attack, the free application of the actual cautery holds out a fair probability of checking the disease; but, when once formed, free and deep incisions are the only treatment on which any reliance can be placed.

*Dublin Journal. Jan.* 1840.

